# Mitochondrial transfer/transplantation in lung injury: mechanism, therapeutic potential, and clinical application

**DOI:** 10.3389/fimmu.2025.1668281

**Published:** 2025-09-16

**Authors:** Ling-Jie Wang, Peng-Fei Guo, SongOu Zhang, Sai Wang, Yi-Zhao Chen, Hong-Wang Yan, Xue-Lin Zhang

**Affiliations:** ^1^ Department of Thoracic Surgery, Wenling First People’s Hospital, Wenling Hospital Affiliated to Wenzhou Medical University, Wenling, Zhejiang, China; ^2^ Beijing Century Altar Hospital, Beijing, China; ^3^ School of Medicine, Ningbo University, Ningbo, Zhejiang, China; ^4^ Department of Thoracic Surgery, Jining Third People’s Hospital, Jining, Shandong, China

**Keywords:** lung injury, mitochondrial transfer, ARDS, COPD, ALI

## Abstract

Lung injury has become a critical clinical problem that urgently requires resolution due to its high morbidity, high mortality, and the limitations of existing treatment methods. Mitochondrial dysfunction, as the core mechanism of lung injury, promotes disease progression through energy metabolism imbalances, oxidative stress, and exacerbated inflammatory responses. Recent studies have found that intercellular mitochondrial transfer, acting as a “transcellular rescue” mechanism, can deliver functional mitochondria through pathways such as tunneling nanotubes, exosome. This process provides a novel approach to replenish energy for damaged cells, regulate inflammation, and repair tissues. In various lung injury models, mitochondrial transfer/transplantation has been shown to improve alveolar-capillary barrier function, reduce collagen deposition, inhibit the release of inflammatory factors, and restore mitochondrial membrane potential. This is particularly evident in conditions such as acute lung injury, pulmonary fibrosis, acute respiratory distress syndrome, and chronic obstructive pulmonary disease, where it shows significant therapeutic potential. The combination of diverse delivery methods and multi-source mitochondria provide a flexible strategy for clinical application. In summary, mitochondrial transfer, as an emerging intercellular communication and rescue mechanism, provides a promising new direction for the precision treatment of lung injury.

## 1 Introduction

Lung injury is a pathological process caused by multiple factors, including infection, inflammation, ischemia, toxic exposure, and mechanical stress. Based on the progression of the disease, it can be divided into two categories: acute lung injury (ALI)/acute respiratory distress syndrome (ARDS) and chronic lung injury (such as pulmonary fibrosis, chronic obstructive pulmonary disease (COPD) (1–3). These diseases not only significantly affect patients’ quality of life but also impose a substantial medical burden (4–6). ARDS is one of the leading causes of death in intensive care units, with a mortality rate of up to 40%, which is even higher in elderly and immunocompromised individuals (7). The pathological features of ARDS include alveolar-capillary barrier destruction, pulmonary edema, inflammatory cell infiltration, and diffuse alveolar damage. Currently, standard treatments for ARDS mainly include mechanical ventilation, glucocorticoids, and anti-inflammatory drugs (8). However, these methods only alleviate symptoms and cannot reverse the underlying damage to lung tissue. For example, while mechanical ventilation can sustain oxygenation, prolonged use may lead to ventilator-associated lung injury and exacerbate alveolar damage. Although glucocorticoids suppress inflammatory responses, their nonspecific immunosuppressive effects can increase the risk of secondary infections, and they have no significant impact on the fibrosis process (5, 9, 10). In addition, biological therapies targeting specific inflammatory factors have shown limited effects in some patients, highlighting the heterogeneous nature of ARDS and the need for more precise treatment strategies (11). Representative diseases of chronic lung injury include idiopathic pulmonary fibrosis(IPF) and COPD. IPF is a progressive, irreversible interstitial lung disease, with a median survival of only 3–5 years (12). Its pathological features include the abnormal activation of fibroblasts and excessive deposition of extracellular matrix, which ultimately leads to the destruction of lung structure and loss of function. Although anti-fibrotic drugs can slow disease progression, they cannot repair existing scar tissue, and some patients discontinue the medication due to gastrointestinal side effects (13, 14). COPD is a disease characterized by persistent airflow limitation, with major pathological changes, including alveolar wall destruction and airway remodeling. It is the third leading cause of death worldwide, and currently, no cure exists (15). Existing treatments primarily relieve symptoms but do not prevent disease progression. Patients with COPD often experience mitochondrial dysfunction, manifested as reduced ATP production and excessive ROS production, which may contribute significantly to disease progression (16–18). Despite recent advancements in the treatment of lung injury, existing approaches still face numerous challenges. Traditional anti-inflammatory therapies, such as glucocorticoids, can reduce inflammation by broadly suppressing immune responses. However, their non-specific mechanisms of action lead to severe side effects, including osteoporosis, diabetes, and an increased risk of infection with long-term use. Additionally, their effect on slowing pulmonary fibrosis progression is limited, suggesting that inflammation and fibrosis may drive disease progression through distinct mechanisms (19, 20). In the field of stem cell therapy, although mesenchymal stem cells (MSCs) are highly expected for their immunomodulatory and tissue repair abilities, clinical outcomes have shown significant limitations. In addition, the standardization and heterogeneity of stem cell preparations have led to inconsistent results in clinical trials (21–23). In gene and cell therapy, while CRISPR and mRNA-based repair strategies theoretically offer high specificity, they face significant obstacles such as low delivery efficiency and insufficient targeting in practical applications. These limitations have prompted researchers to explore new therapeutic targets. As a key mechanism of lung injury, mitochondrial dysfunction has garnered increasing attention in recent years due to its core role in key pathological processes such as energy metabolism, oxidative stress, and apoptosis. This has opened a new avenue for developing more effective treatments for lung injury (24, 25).

Mitochondria are not only the “energy factories” of cells but also play crucial roles in calcium homeostasis, reactive oxygen species (ROS) regulation, and programmed cell death. In lung injury, mitochondrial dysfunction is a key factor driving disease progression. This dysfunction involves multiple mechanisms, including mitochondrial quality control imbalances, mtDNA mutations, and ROS bursts. Mitochondria maintain their functional integrity through dynamic balance and selective autophagy, processes that are crucial for the homeostasis of alveolar epithelial cells (AECs) and pulmonary microvascular endothelial cells (PMVECs) in lung tissue (25, 26). Studies have shown that in the lung epithelial cells of patients with IPF, the level of mitochondrial autophagy mediated by the PARK2 pathway is significantly reduced, leading to abnormal mitochondrial accumulation and excessive collagen secretion (27). This suggests that defects in mitochondrial autophagy could be an important pathogenic mechanism and therapeutic target in pulmonary fibrosis (28). At the same time, mitochondrial dynamics imbalance also plays a key role in lung injury. Disruptions in mitochondrial fission and fusion can aggravate the pathological process (29). For example, in the LPS-induced ALI model, Drp1 inhibitors (such as Mdivi-1) can reduce mitochondrial fragmentation and improve endothelial barrier function. Additionally, promoting mitochondrial fusion has been shown to significantly enhance the anti- apoptotic ability of AECs (30). These findings collectively highlight the core regulatory role of mitochondrial quality control mechanisms in lung injury. Mitochondrial DNA (mtDNA) is particularly vulnerable due to the absence of histone protection and its limited repair capacity, making it more susceptible to damage under oxidative stress. As a result, the level of free mtDNA in the plasma of patients with ARDS is significantly elevated, which can serve as a biomarker of disease severity (31). Once mtDNA is released into the cytoplasm, it can aggravate the inflammatory response by activating the cGAS-STING pathway or TLR9 signaling (32). Cx43 plays a crucial role in maintaining mitochondrial health. Cx43 forms a hemichannel with a conductance of ~130 pS in the inner submitochondrial membrane (33), maintaining ion homeostasis by regulating K^+^/Ca²^+^ flux. Its open probability is finely regulated by phosphorylation sites such as S262/S368. Inhibiting Cx43 significantly reduces the risk of calcium-triggered mitochondrial permeability transition pore opening (34). Cx43 can directly enhance complex I activity and improve oxidative phosphorylation efficiency (33). It also mediates mitochondrial transfer to remodel nucleoside metabolism in leukemic stem cells (35). Furthermore, in lung injury, Cx43 enables intercellular transfer of functional mitochondria via TNTs (36). The phosphorylation status of Cx43 determines the sensitivity of mPTP during cardiac ischemia (34), while lung tissue relies on its-mediated mitochondrial transfer to repair epithelial energy metabolism (36). These findings indicate that Cx43 is an important hub for regulating mitochondrial dynamic balance.

Traditionally, it’s believed that cells can only repair their own mitochondria through autophagy or biosynthesis. However, recent studies have revealed that mitochondria can actively transfer between cells, forming a “transcellular rescue” network (37). This discovery provides a new perspective for the treatment of lung injury. In 2012, Islam et al. demonstrated that bone marrow-derived MSCs (BM-MSCs) can transfer functional mitochondria to AECs via TNTs, thereby rescuing cell death caused by ATP deficiency (38). In addition to MSC-mediated mitochondrial transfer, mitochondria-rich tissues, such as muscle and liver, can also serve as mitochondrial donors, while damaged AECs and airway epithelial cells are the primary recipients (39). In the LPS-induced ALI model, MSC-derived mitochondria can be transferred to epithelial cells through vesicles or direct contact, restoring their oxidative phosphorylation capacity (40). Based on the discovery of mitochondrial transfer, researchers have proposed the possibility of extracting mitochondria *in vitro* and using mitochondrial transplantation as a therapeutic strategy for diseases. In a bleomycin-induced pulmonary fibrosis model, intravenous injection of exosomes containing functional mitochondria was shown to reduce collagen deposition (41). This suggests that mitochondrial transfer has great research potential in the treatment of lung injury diseases. This review discusses the current state of research on mitochondrial transfer and delivery in lung injury, as well as future directions in this promising field.

## 2 Biological mechanisms of mitochondrial transfer

As an emerging method of intercellular communication, the biological mechanism of mitochondrial transfer involves a complex structural foundation, cell interactions, and a molecular regulatory network (42). In recent years, advancements in super-resolution microscopy and *in vivo* imaging technologies have allowed researchers to gradually uncover the intricate regulatory mechanisms behind this process.

Mitochondrial transfer between cells occurs through multiple pathways, each with distinct mechanisms. One of the main pathways involves TNTs, which are cell membrane extensions with a diameter of 50–1000 nm. These TNTs can establish intercellular connections over distances that span multiple cell diameters in the microenvironment (43). TNTs are rich in F-actin, and their stability relies on the activation of Rho GTPase family proteins (44, 45). Miro1/2, as mitochondrial outer membrane anchor proteins, coordinate the directional migration of mitochondria along microtubules to the TNTs transport site through kinesin (46). Rac1 promotes actin polymerization by activating the WAVE complex, driving the cell membrane protrusion to form the physical structure of the TNTs (47). These mechanisms work together to optimize the efficiency of mitochondrial transfer between cells.

**Figure 1:**
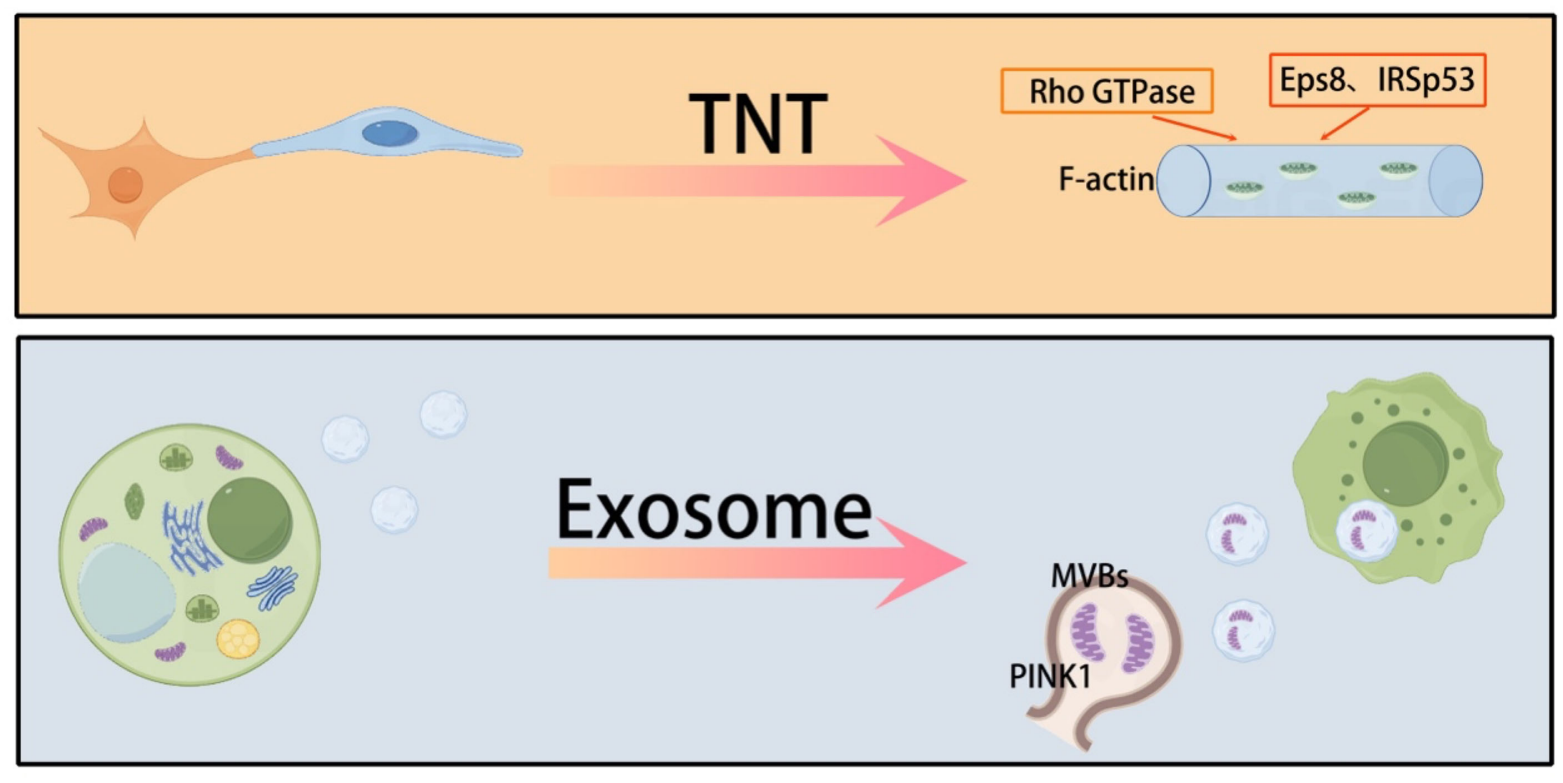
The main ways of mitochondrial transfer between cells.

Studies have found that Eps8/IRSp53-dependent linear actin polymerization promotes the formation of TNTs. High-resolution electron microscopy shows that TNTs contain track proteins necessary for transporting organelles, such as mitochondria and the endoplasmic reticulum. Besides TNTs, exosomes (30–150 nm) and microvesicles (100–1000 nm), which are major subpopulations of extracellular vesicles (EVs), also mediate the delivery of mitochondrial components in lung injury. Mitochondria detached from the respiratory chain recruit phosphatase and tensin homolog-induced putative kinase 1 (PINK1) to the outer mitochondrial membrane. Subsequently, mitochondria expressing PINK1 interact with late endosomes/multivesicular bodies, which release small EVs after fusion with the plasma membrane. These exosomes are enriched with mitochondria and mtDNA fragments (48, 49). Recipient cells can take up mitochondria-containing exosomes via endocytosis. However, the molecular mechanisms behind the release of mitochondrial components from EVs once internalized by recipient cells remain unclear (50). The membrane components of EVs, including phospholipids, integrins, and tetraspanins, may facilitate this uptake by promoting membrane fusion or engaging in the endocytic pathway (50). Some believe that gap junctions are another pathway for mitochondrial transfer (51). However, this view remains controversial. Gap junction pores only allow the transfer of molecules with a molecular weight less than 2 kDa. Therefore, gap junctions may not be able to achieve mitochondrial transfer. In addition, cell fusion, while less common, is an efficient mitochondrial transfer method that relies on the activation of fusogenic proteins (such as syncytin-1) and phospholipase D (52). Together, these diverse transfer pathways form a complex intercellular mitochondrial communication network, providing a multi-level regulatory mechanism for lung injury repair. Mitochondrial transfer presents a cell-specific regulatory pattern, with MSCs being the primary mitochondrial donors (53). The CXCL12/CXCR4 chemokine axis plays a key role in this process (54), promoting the migration of MSCs to the injury site and facilitating their migration. Gene editing experiments have confirmed that knocking out CXCR4 can reduce the efficiency of MSC migration (55). An intriguing “energy selectivity” phenomenon has been observed in mitochondrial transfer (56, 57). Unlike the unidirectional transfer of MSCs, macrophages and endothelial cells maintain a dynamic equilibrium with bidirectional mitochondrial exchange between them (53, 58). At the molecular regulatory level, mitochondrial movement-related genes such as Miro1 and KIF5B directly activate this process (59–61), with CD38 acting as a regulator. The use of CD38 inhibitors or shRNA treatment can restore the mitochondrial transfer ability (62). These findings highlight the precise molecular regulatory network involved in mitochondrial transfer and provide a theoretical basis for developing targeted therapeutic strategies (**Figure 1**). 

TNTs and exosomes are the main means of transferring mitochondria between cells.

## 3 Mitochondrial transfer/transplantation and its therapeutic potential in lung injury repair

Mitochondrial transfer/transplantation has shown significant therapeutic potential in the repair of lung injury across various disease models. These findings not only confirm the biological effects of mitochondrial transfer/transplantation but also provide insights into its mechanisms of action, laying a strong foundation for clinical application.

### 3.1 ALI

ALI is characterized by the destruction of the alveolar-capillary barrier, inflammatory infiltration, and oxidative stress, with mitochondrial dysfunction playing a key role in its pathogenesis. Studies have demonstrated that mitochondrial transfer can effectively improve the pathological processes associated with ALI. In the LPS-induced ALI model, MSCs are identified as key donors for mitochondrial transfer. Both lung tissue-derived MSCs and bronchoalveolar lavage fluid (BALF)-derived MSCs can transfer mitochondria to bronchial epithelial cells (BEAS2B) via TNTs or microvesicles. Importantly, the transfer efficiency between these MSCs and BM-MSCs is comparable, suggesting that endogenous lung MSCs can play an active role in the repair of ALI through mitochondrial transfer (63). In addition, exogenous airway instillation of BM-MSCs can enhance the repair effect of lung injury via local mitochondrial transfer (38). Recent advances in medical materials have further optimized mitochondrial transfer. For example, BMSCs modified with selenium nanoparticles (SeNPs@CS) have been shown to upregulate miR-20b, enhancing their ability to transfer healthy mitochondria to damaged AECs type II (AECII). This transfer reduces intracellular ROS levels, restores ATP generation, and significantly reduces cell apoptosis (64). Additionally, mitochondrial transfer in this context has been shown to reduce Th17 cell differentiation (lowering IL-17 levels) by inhibiting the RORγt/STAT3 signaling axis and to upregulate the anti-inflammatory factor IL-10, thereby reducing lung tissue inflammatory infiltration (64). In an LPS-induced pulmonary microvascular endothelial cell (PMVEC) injury model, MSCs regulated TNTs formation through mitochondrial transcription factor A (TFAM), enhancing mitochondrial transfer to PMVECs. This results in increased transmembrane resistance, decreased lung wet/dry weight ratios, and reduced Evans blue dye exudation, indicating improved vascular permeability (65). Further experiments in the mouse lung ischemia/reperfusion (I/R) model showed that both pulmonary artery injection and aerosol inhalation of mitochondria can improve lung dynamic compliance, reduce resistance, and significantly reduce neutrophil infiltration and cell apoptosis, suggesting that mitochondrial transplantation can improve lung mechanical function by protecting vascular endothelial and alveolar structures (66). Mitochondrial transplantation has also been shown to reduce oxidative stress damage by upregulating the expression of antioxidant enzymes. In endotoxin-induced ALI rats, allogeneic mitochondrial transplantation significantly increased the activities of superoxide dismutase and glutathione peroxidase in lung tissue, decreased malondialdehyde (MDA) levels, and increased arterial oxygen tension from 78.9 mmHg to 95.9 mmHg. These changes were accompanied by a reduction in protein content in bronchoalveolar lavage fluid (BALF), indicating repair of the alveolar-capillary barrier (67). Similar results were observed in the bleomycin-induced lung injury model, where mitochondrial transplantation increased glutathione levels, decreased MDA, and improved mitochondrial membrane potential and succinate dehydrogenase activity, further supporting its antioxidant and mitochondrial function protection effects (68). For sepsis-related ALI, in the mouse cecal ligation and puncture model, intravenous injection of muscle-derived mitochondria improved survival rates in septic mice, enhanced bacterial clearance, reduced levels of proinflammatory factors (IL-6, IL-1β), and alleviated damage to multiple organs, including the lung, liver, and kidneys. The underlying mechanism may involve the regulation of extracellular matrix receptor interactions and the complement-coagulation cascade (39). When comparing mitochondrial sources, a pig *ex vivo* lung perfusion (EVLP) experiment found that xenogeneic, autologous, and allogeneic mitochondrial transplantation all significantly improved the P/F ratio, reduced pulmonary artery peak pressure, and did not cause acute immune rejection. These results suggest that xenogeneic mitochondria could serve as a “ready-made” source to reduce ischemia-reperfusion injury in transplanted lungs (69).

These findings collectively highlight the multi-target regulatory advantages of mitochondrial transfer in ALI treatment. Mitochondrial transfer/transplantation not only improves energy metabolism by directly supplying functional mitochondria but also protects lung tissue structure by modulating inflammation and oxidative stress. Therefore, mitochondrial transfer could become a valuable complement to traditional anti-inflammatory and antioxidant treatments, providing additional options for clinical management of lung injury (**Table 1**).

**Table 1:** Summary of research on mitochondrial transfer in ALI.

Transplantation/transfer method	Interventions	Key findings	Animal models	Cell Model	PMID
BMSC-mediated transfer	Airway instillation of BMSC	BMSCs can prevent ALI by restoring alveolar bioenergetics through Cx43-dependent alveolar attachment and mitochondrial trafficking	LPS-induced acute lung injury	None	22504485
Intravenous injection or airway aerosolization	Muscle-derived healthy mitochondria	Mitochondrial transplantation via vascular delivery or nebulization improves lung mechanics and reduces lung tissue damage.	Ischemia-reperfusion-induced lung injury	None	31693391
BMSC-mediated mitochondrial transfer	Tail vein injection of SeNPs@CS optimized BMSCs	SeNPs@CS-optimized BMSCs reduced the levels of inflammatory markers in a mouse model, showing superior efficacy over conventional BMSC therapy	Sepsis-induced lung injury	LPS-treated AECII cells	40114196
TNTs	TFAM gene knockdown in MSCs	Controlling the expression of TFAM in MSCs plays a key role in TNTs-mediated mitochondrial transport to improve the permeability barrier of PMVECs.	LPS-induced lung injury in mice	LPS-induced PMVEC	37060506
Tail vein injection	Muscle-derived healthy mitochondria	Replenishing mitochondria can reduce systemic inflammation and organ damage, enhance bacterial clearance, and improve survival in patients with sepsis.	Sepsis-induced lung injury	None	33060455
Tail vein injection	Muscle-derived healthy mitochondria	Exogenous mitochondria can inhibit GSH depletion, reduce MDA production, increase SDH activity, and prevent MMP loss and histopathological abnormalities	BLM-induced lung injury in rats	None	40063258
Jugular vein injection	Muscle-derived healthy mitochondria	Living mitochondrial transplantation preserves the integrity of the alveolar-capillary barrier endothelium and improves gas exchange during the acute phase of endotoxin-induced acute lung injury	LPS-induced lung injury in mice	None	35813297
Airway instillation	Mitochondria from liver and muscle	Mitochondrial transplantation increased the P/F ratio of the lungs during EVLP and reduced the peak pulmonary artery pressure compared with lungs without mitochondrial transplantation.	Ischemia-reperfusion-induced lung injury	None	39536924
TNTs and microvesicles	Co-culture with stem cells	LT-MSCs and BAL-MSCs can donate cytoplasmic contents and mitochondria to bronchial epithelial cells through multiple mechanisms	None	BEAS2B epithelial cells	27406134

### 3.2 Pulmonary fibrosis

Research on mitochondrial transfer/transplantation in pulmonary fibrosis has shown that regulating mitochondrial homeostasis, improving mitochondrial function, and removing damaged mitochondria can effectively alleviate the fibrotic process. These findings provide a promising new direction for the treatment of pulmonary fibrosis. At the mechanistic level, multiple studies have revealed the close relationship between mitochondrial dysfunction and pulmonary fibrosis. Mitochondrial dysfunction in AECs is a key pathological factor in the progression of pulmonary fibrosis. For instance, Pioglitazone has been shown to activate the PGC1α/NRF1/TFAM pathway, promoting mitochondrial biogenesis in MSCs. Furthermore, iron oxide nanoparticles can enhance mitochondrial delivery, improving the efficiency of mitochondrial transfer. This intervention has been shown to restore mitochondrial function in mouse models to 107.8% of normal levels, activate damaged mitochondrial autophagy, and reduce the accumulation of dysfunctional mitochondria (70). Abnormal mitochondrial iron metabolism is one of the key therapeutic targets in mitochondrial therapy for pulmonary fibrosis. In bleomycin-induced pulmonary fibrosis, the mitochondrial iron transporter MFRN2 is highly expressed in AECs type II (AECII), leading to iron deposition and mitochondrial dysfunction. Activation of the EP4 receptor can promote the ubiquitination and degradation of IREB2 by upregulating FBXL5, thereby inhibiting MFRN2-mediated iron accumulation and improving mitochondrial function (71). Recent advances in cell engineering and nanotechnology have further enhanced mitochondrial transfer efficiency. The Mito-MEN system, developed by Wang et al., uses nanoparticles to deliver Parkin mRNA, improving the efficiency of mitochondrial transfer to damaged cells. This system not only promotes Parkin-mediated mitochondrial autophagy but also reduces collagen deposition and TGF-β secretion in a bleomycin-induced mouse model, leading to a reduction in pulmonary fibrosis scores (72). Mitochondria derived from human umbilical cord MSCs have also been shown to inhibit TGF-β-induced fibroblast activation and epithelial-mesenchymal transition *in vitro*. *In vivo*, intravenous injection of these mitochondria in a bleomycin-induced mouse model reduced lung weight gain, inflammatory cell infiltration, and Ashcroft fibrosis scores (41). Mitochondrial transplantation operates through multiple mechanisms: it replenishes functional mitochondria, restores ATP production, reduces ROS levels, and mitigates cell damage by regulating mitochondrial autophagy and iron metabolism (71, 73).Additionally, it inhibits myofibroblast activation and excessive extracellular matrix deposition (41). In summary, mitochondrial transplantation has shown significant potential in the intervention of pulmonary fibrosis by enhancing the supply of functional mitochondria, clearing damaged mitochondria, and regulating key metabolic pathways. These findings provide a solid experimental foundation for the development of therapeutic strategies aimed at restoring mitochondrial homeostasis in pulmonary fibrosis (**Table 2**).

**Table 2:** Summary of research on mitochondrial transfer in IPF.

Transplantation/transfer method	Interventions	Key findings	Animal models	Cell Model	PMID
BMSC-mediated mitochondrial transfer	Pioglitazone and iron oxide nanoparticles combined treatment of BMSCs	Increasing the number of mitochondria in hMSCs can enhance mitochondrial transfer capacity	BLM-induced PF model in mice	Organoid Models	37723135
Tail vein injection	Add mitochondria to cell culture medium	Healthy mitochondria rescue BLM-lesioned AECII loss of mitochondrial iron deposition and cellular damage.	BLM-induced PF model in mice	BLM-induced damage in MLE-12 cells	38773980
Tail vein injection	Mito-MEN anchors isolated healthy mitochondria	Mito-MEN increases the load of functional exogenous mitochondria by enhancing mitochondrial delivery efficiency and promotes mitophagy of dysfunctional endogenous mitochondria	BLM-induced PF model in mice	None	39546755
Tail vein injection	Mitochondria from umbilical cord mesenchymal stem cells	Intravenous injection of umbilical cord mesenchymal stem cell-derived mitochondria reduces pulmonary fibrosis	BLM-induced PF model in mice	TGF-treated human lung fibroblasts and human bronchial epithelial cells	39684495

### 3.3 ARDS

ARDS is an acute lung disease characterized by alveolar-capillary barrier destruction, uncontrolled inflammation, and oxidative stress, with a high mortality rate. Mitochondrial transfer/transplantation presents a promising new potential direction for its treatment. Mitochondrial transfer/transplantation plays a key role in immune regulation in ARDS. Studies have shown that alveolar macrophages are the core mediators of mitochondrial transfer, which allows them to exert both antibacterial and anti-inflammatory effects. If macrophages are depleted, the bacterial clearance ability of MSCs is completely impaired (74). When macrophages acquire mitochondria, their phagocytic ability is significantly enhanced, enabling them to clear bacteria more effectively. Moreover, mitochondrial acquisition leads to macrophage polarization toward the anti-inflammatory M2 phenotype, characterized by the upregulation of M2 markers such as CD206 and the reduced secretion of pro-inflammatory cytokines like TNF-α and IL-6. This, in turn, helps inhibit excessive inflammatory responses (49, 74, 75). In terms of mitochondrial repair of lung tissue, mitochondrial dysfunction in pulmonary microvascular endothelial cells and airway epithelial cells plays a significant role in ARDS pathology. The resulting loss of mitochondrial function leads to increased barrier permeability. By supplementing functional mitochondria, it is possible to restore mitochondrial respiratory function in these cells, improve ATP production, stabilize membrane potential, reduce ROS accumulation, and thus reduce cellular permeability and help repair the alveolar-capillary barrier (40). Additionally, mitochondria play a role in balancing mitochondrial autophagy and biogenesis, preventing excessive autophagy from causing cellular damage, and promoting the restoration of lung tissue homeostasis (40). *In vivo* experiments further support the therapeutic potential of mitochondrial transfer/transplantation in ARDS. In mouse models of ARDS induced by LPS or bacteria, mitochondrial transplantation significantly reduced inflammatory cell infiltration, lowered protein content and pro-inflammatory factor levels in BALF, and alleviated lung tissue damage (74, 75). Furthermore, transplanting alveolar macrophages pre-treated with mitochondria-containing EVs significantly reduced inflammation in lung tissue and decreased neutrophil infiltration (49). The therapeutic effect of mitochondrial transplantation is also influenced by the inflammatory phenotype of patients with ARDS. The reparative effect is more pronounced in patients with a low-inflammatory phenotype, whereas it is more limited in high-inflammatory environments (40) (**Table 3**).

**Table 3:** Summary of research on mitochondrial transfer in ARDS.

Transplantation/transfer method	Interventions	Key findings	Animal models	Cell Model	PMID
extracellular mitochondria	Treatment with exosomes with or without mitochondria	MSC-EVs improve alveolar capillary barrier properties by restoring mitochondrial function, at least in part through mitochondrial transfer.	LPS-induced acute respiratory distress syndrome	Human small airway epithelial cells and pulmonary microvascular endothelial cells and human precision-cut lung sections	33334945
extracellular mitochondria	Exosomes containing EVs intervene in macrophages	In the setting of ARDS, MSCs promote an anti-inflammatory and highly phagocytic macrophage phenotype via EV-mediated mitochondrial transfer.	LPS-induced acute respiratory distress syndrome	LPS-treated macrophages	28598224
Tail vein injection	Human stem cell-derived mitochondria	Mitochondria isolated from human stem cells have anti-inflammatory effects on alveolar macrophages and anti-apoptotic effects on alveolar epithelial cells	ARDS mouse model established by LPS	LPS-treated mouse alveolar macrophages and human pulmonary microvascular endothelial cells	39334020
TNTs	Co-culture with MSCs	Transfer of MSCs to the mitochondria of innate immune cells enhances phagocytic activity	Escherichia coli-induced pneumonia model	Monocytes	27059413

Mitochondrial transfer/transplantation can improve IPF through TGF-β and mitophagy. Mitochondrial transplantation and transfer can promote M2 polarization and regulate TNF-α and IL-6 in ARDS. In ALI, mitochondrial transplantation and transfer can inhibit cell apoptosis and suppress oxidative stress.

### 3.4 Other lung diseases

COPD, asthma, airway hyperresponsiveness, and obesity-related allergic airway inflammation are all common lung conditions, characterized by airway inflammation, structural damage, and functional impairment. Mitochondrial transfer/transplantation shows significant therapeutic potential in these diseases by repairing damaged cells, regulating inflammation, and restoring mitochondrial function. In COPD, induced pluripotent stem cell-derived mesenchymal stem cells (iPSC-MSCs) exhibit stronger mitochondrial transfer ability than BM-MSCs. Studies have demonstrated that iPSC-MSCs can transfer functional mitochondria to airway epithelial cells damaged by cigarette smoke through TNTs, significantly restoring cellular ATP levels and reducing alveolar structural damage and fibrosis (76). The efficiency of this transfer is influenced by the damaged microenvironment, with cigarette smoke extracts promoting this process. In addition, iPSC-MSCs have a higher retention rate in the lungs and demonstrate a more significant repair effect compared to BM-MSCs. For airway hyperresponsiveness, mitochondrial transplantation has been shown to reverse cholinergic hyperactivity induced by the combination of cigarette smoke and lipopolysaccharide. Exogenous mitochondria reduce ROS production in airway epithelial cells, inhibit the sensitivity of M3 receptors, reduce the contractile response of the airway to acetylcholine, and improve the frequency of ciliary beating. These effects help reduce mucus secretion and inflammatory infiltration (77). A key mechanism of this process is mitochondrial transfer mediated by Cx43. iPSC-MSCs can transfer mitochondria to damaged airway epithelial cells through TNTs, and overexpression of Cx43 enhances TNTs formation and transfer efficiency. This significantly reduces the release of Th2 cytokines (IL-4, IL-5), restores mitochondrial membrane potential, and alleviates airway inflammation and mucus secretion (78). In contrast, silencing Cx43 impairs the therapeutic effect of mitochondrial transfer. In obesity-related allergic airway inflammation, MSCs derived from obese individuals (MSC-Ob) exhibit impaired mitochondrial transfer capacity due to mitochondrial dysfunction, characterized by cristae damage, ROS accumulation, and reduced cardiolipin content. However, Pyrroloquinoline quinone can enhance the mitochondrial transfer of MSC-Ob to airway epithelial cells by restoring cardiolipin-dependent mitochondrial autophagy. This treatment reduces Th2 cytokine levels and alleviates airway hyperresponsiveness (79). Moreover, when cytochalasin B is used to block TNTs formation, MSC-mediated mitochondrial transfer is completely inhibited (80). *In vivo* experiments have confirmed that mitochondrial transfer improves bacterial clearance and reduces pro-inflammatory factors such as TNF-α and IL-6. This mechanism underlies the antibacterial and anti-inflammatory effects of MSCs. Overall, mitochondrial transfer/transplantation shows therapeutic potential in various lung diseases by repairing damaged cell energy metabolism and regulating inflammatory pathways. The efficiency of mitochondrial transfer/transplantation is influenced by factors such as cell source, microenvironment, and molecular regulation, providing a basis for therapies targeting mitochondrial homeostasis (**Table 4**).

**Table 4:** Summary of research on mitochondrial transfer in other lung disease.

Disease type	Transplantation/transfer method	Interventions	Key findings	Animal models	Cell model	PMID
Allergic airway inflammation	TNTs	obesity	Obesity affects the therapeutic effect of mitochondrial transfer	Allergic asthma mice induced by HDM and Ova	None	37173333
asthmatic inflammation	TNTs	CX43 overexpression or silencing	CX43 plays a key role in regulating TNTs formation by mediating mitochondrial transfer between iPSC-MSCs and epithelial cells	OVA-induced asthma in mice	OVA-treated lung epithelial cells	30344008
Lung inflammation	TNTs	cytochalasin B	When cytochalasin B blocked TNTs formation, the effect of MSCs on macrophage phagocytosis was completely abolished	None	GM-CSF-treated macrophages	28534038
Airway Hyperresponsiveness	Airway instillation	Airway cell-derived mitochondria	Mitochondrial transplantation effectively alleviates airway hyperresponsiveness induced by cigarette smoke and lipopolysaccharide by inhibiting ROS-enhanced epithelial cholinergic hyperactivity	Rats treated with cigarette smoke plus LPS	LPS-induced airway epithelial model	27279915
Pulmonary hypertension	Tail vein injection	Muscle-derived mitochondria	Transplantation of viable mitochondria restores pulmonary artery contractile phenotype and vascular reactivity, resulting in reduced afterload and right ventricular remodeling in rats with established pulmonary hypertension	Pulmonary hypertension induced by monocrotaline	None	32948302
COPD	TNTs	MSC-derived mitochondria	iPSC-MSCs have a higher mitochondrial transfer capacity than BM-MSCs, thereby being able to repair CS-induced mitochondrial damage	Cigarette smoke-induced COPD	BEAS-2B	24738760

## 4 Mitochondrial transfer/transplantation in lung delivery

In studies related to lung diseases, the delivery methods of mitochondria are diverse, reflecting the special characteristics of the lung, which is directly exposed to the external environment and has both airway and vascular pathways. In addition to conventional intravenous injections, several local transfer/transplantation methods are commonly used to target the lungs more precisely. Cell-mediated transfer largely relies on MSCs (BM-MSCs, iPSC-MSCs, etc.) to transfer mitochondria directly to damaged lung cells. These MSCs transfer functional mitochondria to bronchial epithelial cells, AECs, and other lung cell types through various mechanisms, including TNTs, and microvesicles. In non-cell-mediated delivery, a variety of methods have been developed to directly transplantation mitochondria to the lung tissue. Besides intravenous injections, localized methods like airway perfusion, nebulization inhalation, and airway instillation are frequently used for targeted therapy. These approaches allow for precise transplantation to the lung, particularly useful for diseases affecting the airways or alveolar structures. For example, airway perfusion of mitochondria has shown promise in improving lung function during EVLP procedures, while nebulization or airway instillation can effectively treat airway hyperresponsiveness and alveolar damage. Systemic methods, such as pulmonary vascular injection or tail vein injection, are also explored for mitochondrial transplantation, offering broader distribution throughout the lung tissue. The sources of mitochondria for transplantation are varied, offering flexible strategies for mitochondrial therapy. Donor cells such as MSCs from tissues like bone marrow, umbilical cord, adipose, muscle, and liver are common sources of functional mitochondria. Additionally, iPSC-MSCs, and healthy mitochondria isolated directly from muscle, liver, or airway cells, are also used. Among these sources, iPSC-MSCs are particularly beneficial for repairing mitochondrial damage caused by cigarette smoke because of their stronger mitochondrial transfer ability compared to BM-MSCs. These diverse delivery methods, combined with a variety of mitochondrial sources and the unique anatomical and functional characteristics of the lungs, provide flexible and targeted strategies for mitochondrial-based therapies. This allows for more effective interventions in lung diseases such as pulmonary fibrosis, ALI, ARDS, asthma, and COPD.

## 5 Therapeutic potential and translational strategy

Mitochondrial transfer/transplantation offers unique therapeutic advantages and translational prospects in lung injury repair. Its core value lies in the synergistic effects of multi-target regulation and tissue repair. In terms of therapeutic potential, this mechanism can simultaneously improve the energy metabolism of damaged cells, inhibit oxidative stress, and regulate the inflammatory response by directly supplementing functional mitochondria, achieving a “two birds with one stone” therapeutic effect. In the ALI model, mitochondria derived from MSCs can repair the alveolar epithelial barrier; in the pulmonary fibrosis model, mitochondrial transplantation can reduce collagen deposition; in COPD, iPSC-MSCs can reverse airway damage caused by cigarette smoke. These findings support its broad applicability across various diseases.

**Figure 2:**
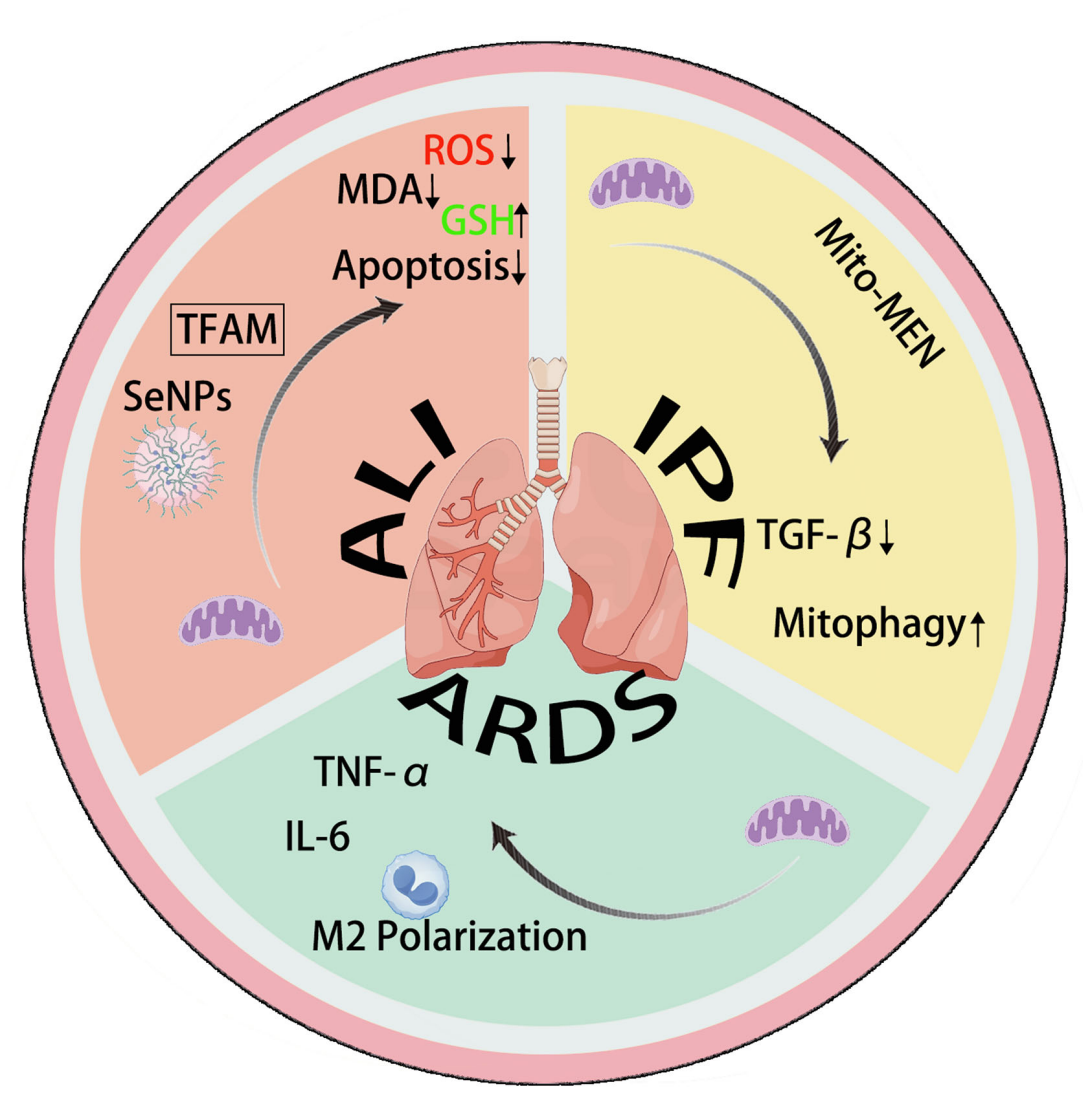
The mechanism of action of mitochondrial transfer in IPF, ALI, and ARDS.

Regarding translational strategies, diverse delivery methods provide flexibility for clinical applications: cell-mediated delivery enhances targeting due to its directional migration ability; non-cell-mediated delivery (such as aerosol inhalation and airway instillation) can directly act on the lung’s lesion site, reducing systemic side effects. The choice of mitochondrial sources is also expanding, including bone marrow, umbilical cord, muscle tissue, and stem cell-derived mitochondria. Among these, iPSC-MSCs show superior potential due to their high transfer efficiency and long retention time. However, key challenges remain in the translational process: standardizing the extraction and quality control of mitochondria, improving delivery efficiency while minimizing the risk of immune rejection, and determining the optimal timing for intervention at different disease stages. With advancements in *in vivo* imaging technologies and nanocarrier engineering, mitochondrial transfer is expected to move from basic research to clinical practice, offering a new precision therapy that could overcome current treatment limitations (**Figure 2**).

## 6 Critical discussion

Existing research has primarily focused on the active role of mitochondrial transfer in repairing damaged tissues. However, recent studies have shown that active mitochondrial transfer also occurs within the tumor microenvironment, potentially supporting cancer cells’ energy metabolism, promoting tumor growth, invasion, and immune evasion (81–83). Although there is currently no direct evidence that therapeutic mitochondrial transfer can induce or promote the development of lung tumors, it is a potential risk that cannot be ignored. Long-term tumorigenicity assessment must be an integral component of safety evaluation in future preclinical and clinical studies. When transplanted mitochondria are derived from non-homologous donors, both the donor and the recipient’s own mtDNA will coexist in the recipient cells, forming a heteroplasmic state (84). The respiratory chain subunits encoded by the donor and recipient mtDNA may not be fully compatible, affecting the assembly and function of the respiratory chain complex (84). Heterogeneous mtDNA may be recognized as “non-self” by the recipient’s immune system, inducing a potential immune response. Although xenogeneic mitochondrial transplantation did not cause acute rejection in a lung injury model, this does not mean that long-term or systemic immune risks do not exist (69). Stringent screening of donor mitochondrial sources, optimization of purification protocols to reduce mtDNA contamination, and development of allogeneic “universal” mitochondrial libraries are potential approaches to mitigate this risk. Transfer/transplantation of functional mitochondria can directly restore ATP levels in recipient cells, improve mitochondrial membrane potential, reduce ROS, and enhance oxidative phosphorylation. However, supplementation of mitochondrial biomass alone may not be sufficient to explain all observed effects. The anti-inflammatory and enhanced phagocytic effects induced by mitochondrial transfer in MSCs or macrophages (40, 80) likely involve mitochondrial-associated molecules acting as signaling molecules to activate specific pathways. Blocking mitochondrial transfer completely abolishes the immunomodulatory effects of MSCs (80), suggesting that the transfer process itself or the signals it carries are crucial. The transferred vector itself is also an important mode of intercellular communication and may transmit independent signals. Current evidence suggests that replenishing functional mitochondrial biomass is essential for therapeutic benefits, especially in rapidly restoring energy metabolism and alleviating oxidative damage. However, the complex immunomodulatory, tissue repair, and homeostatic re-establishment effects are likely to be coordinated by signaling pathways triggered by the transferred mitochondria themselves or their associated molecules. Future studies will require more sophisticated experimental designs (such as using mitochondria with defective respiratory function but intact signaling molecules, or purified mitochondrial signaling molecules) to precisely dissect the relative contributions of the two.

Existing studies have primarily focused on short-term effects. The duration of mtDNA retention within recipient cells remains unclear. Studies have shown that donor mtDNA can persist for several days in co-culture systems (53); however, in the complex *in vivo* environment, its retention may be shorter, potentially being cleared or diluted by the recipient cell’s mitophagy system. Theoretically, long-term retention and expression of its gene products are essential for maintaining respiratory chain function. However, short-term therapeutic benefits may be achieved through the enzymes, metabolites, and signaling molecules carried by mtDNA before it is degraded or diluted. For chronic diseases, repeated treatments or strategies may be required to ensure long-term retention/expression of donor mtDNA. Clarifying the duration of mtDNA retention and its impact on long-term therapeutic efficacy is a key future research direction. Understanding the fate of exogenous mitochondria *in vivo* and the differences in their uptake mechanisms in different pathological settings is fundamental to designing effective, durable, and universally applicable therapeutic strategies. More sensitive tracking technologies are needed for long-term *in vivo* studies.
